# Fine-Scale Analysis Reveals Cryptic Landscape Genetic Structure in Desert Tortoises

**DOI:** 10.1371/journal.pone.0027794

**Published:** 2011-11-21

**Authors:** Emily K. Latch, William I. Boarman, Andrew Walde, Robert C. Fleischer

**Affiliations:** 1 Behavioral and Molecular Ecology Research Group, Department of Biological Sciences, University of Wisconsin, Milwaukee, Wisconsin, United States of America; 2 Center for Conservation and Evolutionary Genetics, National Zoological Park, Washington DC, United States of America; 3 Conservation Science Research and Consulting, Spring Valley, California, United States of America; 4 Walde Research and Environmental Consulting, Atascadero, California, United States of America; Lund University, Sweden

## Abstract

Characterizing the effects of landscape features on genetic variation is essential for understanding how landscapes shape patterns of gene flow and spatial genetic structure of populations. Most landscape genetics studies have focused on patterns of gene flow at a regional scale. However, the genetic structure of populations at a local scale may be influenced by a unique suite of landscape variables that have little bearing on connectivity patterns observed at broader spatial scales. We investigated fine-scale spatial patterns of genetic variation and gene flow in relation to features of the landscape in desert tortoise (*Gopherus agassizii*), using 859 tortoises genotyped at 16 microsatellite loci with associated data on geographic location, sex, elevation, slope, and soil type, and spatial relationship to putative barriers (power lines, roads). We used spatially explicit and non-explicit Bayesian clustering algorithms to partition the sample into discrete clusters, and characterize the relationships between genetic distance and ecological variables to identify factors with the greatest influence on gene flow at a local scale. Desert tortoises exhibit weak genetic structure at a local scale, and we identified two subpopulations across the study area. Although genetic differentiation between the subpopulations was low, our landscape genetic analysis identified both natural (slope) and anthropogenic (roads) landscape variables that have significantly influenced gene flow within this local population. We show that desert tortoise movements at a local scale are influenced by features of the landscape, and that these features are different than those that influence gene flow at larger scales. Our findings are important for desert tortoise conservation and management, particularly in light of recent translocation efforts in the region. More generally, our results indicate that recent landscape changes can affect gene flow at a local scale and that their effects can be detected almost immediately.

## Introduction

Features of the landscape can either restrict or promote movements of individuals in natural populations and consequently influence the degree of genetic connectivity among populations. Characterizing the effects of landscape features on genetic variation is essential for understanding how landscapes shape patterns of gene flow and spatial genetic structure of populations, and this objective forms the basis of landscape genetics [1], [2]. Novel approaches in landscape genetics have yielded new insights about how geographic and environmental features structure genetic variation, especially at fine spatial scales, insights that have had implications for ecology, evolution, and conservation biology.

Detecting cryptic population genetic structure and identifying features of the landscape that influence gene flow are central to understanding local- and regional-scale movements and barriers. Decomposing a genetic mixture of samples into their component, panmictic subpopulations (with or without spatial location information) helps to identify genetic discontinuities that could restrict gene flow among populations (e.g. [3], [4], [5], [6]). Both natural (e.g., rivers, mountains; [7], [8], [9], [10] and anthropogenic (e.g., roads, urban areas; [8], [11], [12] barriers that correlate with genetic discontinuities have been identified within landscapes, and recent studies have distinguished among alternative barrier scenarios [13], [14]. Understanding patterns of movement throughout a landscape and environmental features that influence those movements has been particularly useful in population conservation and management, to identify and prioritize populations for action and to design effective management strategies.

Most landscape genetics studies focus on patterns of movements of individuals at a regional scale. For example, some of the best-known studies in landscape genetics of vertebrates (Columbia spotted frogs, [7]; wolverines, [15]; roe deer, [8]; and black bears, [13]) have characterized patterns of gene flow and genetic structure over several thousand to several hundred thousand square kilometers. These studies have provided unique insight into large-scale landscape features and environmental conditions that influence gene flow at a regional scale. However, organisms can also respond to the landscape at a smaller scale, perceiving slight changes in the local landscape and making movement decisions based on these perceptions. Although it is unclear whether factors influencing the movement of individuals at fine spatial scales may be important predictors of genetic structure at broader scales, recent research suggests that they may not [16]. Thus, the genetic structure of populations at a local scale may be influenced by a unique suite of landscape variables that have little bearing on connectivity patterns observed at broader spatial scales. These local effects may be particularly pronounced in harsh environments, where habitat suitability varies considerably over small distances [17], or for organisms that see habitats as coarse- versus fine-grained.

We carried out a landscape genetics study on the desert tortoise (*Gopherus agassizii*) to investigate fine-scale spatial patterns of genetic variation and gene flow in relation to features of the landscape. The desert tortoise occupies portions of three major North American deserts (Sonoran, Mojave, and Colorado; though the Sonoran desert tortoise has recently been proposed as a new species, *Gopherus morafkai*, [18]), and is federally listed as threatened in the northwestern one-third of its geographic range (the ‘Mojave population’, including all tortoises north and west of the Colorado River; [19]). At a broad scale, desert tortoises are separated by the Colorado River, and long-term restricted gene flow between these regions has led to substantial morphological and genetic differences between Sonoran and Mojave desert tortoises [20], [21], [22], [23], [24].

Within the threatened Mojave population, gene flow among populations appears to be influenced primarily by geographic distance [24], [25], [26], [27], [28]. Indeed, the desert tortoise has been characterized as “perhaps the ideal organism for the IBD [isolation by distance] model; one that is distributed across the landscape in isolated patches and for which the difficulty of dispersal is a function of distance” [26]. This may be true at a regional scale, but makes little sense at a local scale, where desert tortoises make daily movement decisions based in part on landscape features such as elevation, slope, brush cover, and water availability. Desert tortoises are familiar with the locations of resources within their home ranges, including cover sites, mates, forage, mineral licks, and drinking sites, and can travel to these resources for several hundred meters [29]. In addition, the microhabitat within a burrow is significantly cooler and more humid than the surrounding environment [30], perhaps encouraging aggregation of tortoises around optimal burrow sites. These characteristics indicate that desert tortoises may be locally aggregated in accordance with landscape variables at a finer scale than has previously been investigated.

Local landscape effects on desert tortoise gene flow would be obscured if sampling and analyses were performed at a regional scale, where the main force driving patterns of genetic structure is probably geographic distance. Thus, fine-scale characterization of genetic structure is the most relevant for understanding the influence of the landscape on gene flow in the desert tortoise. This study had two main objectives. The main aim of this study was to assess the pattern of gene flow at a fine scale, where several potential barriers to local gene flow exist. In general, we expected to find low levels of population substructure within a local population due to the fairly high population density in the study area and high dispersal potential for desert tortoises. Our second goal was to characterize the extent to which landscape variables, such as elevation, slope, roads, habitat discontinuities, and power lines, would explain desert tortoise gene flow compared to a standard isolation-by-distance model. The integration of molecular genetic techniques with spatial analyses will provide unique insight into landscape genetics at a fine-scale, which in turn will permit more efficient conservation and management of tortoises in this region.

## Results

**Figure 1:**
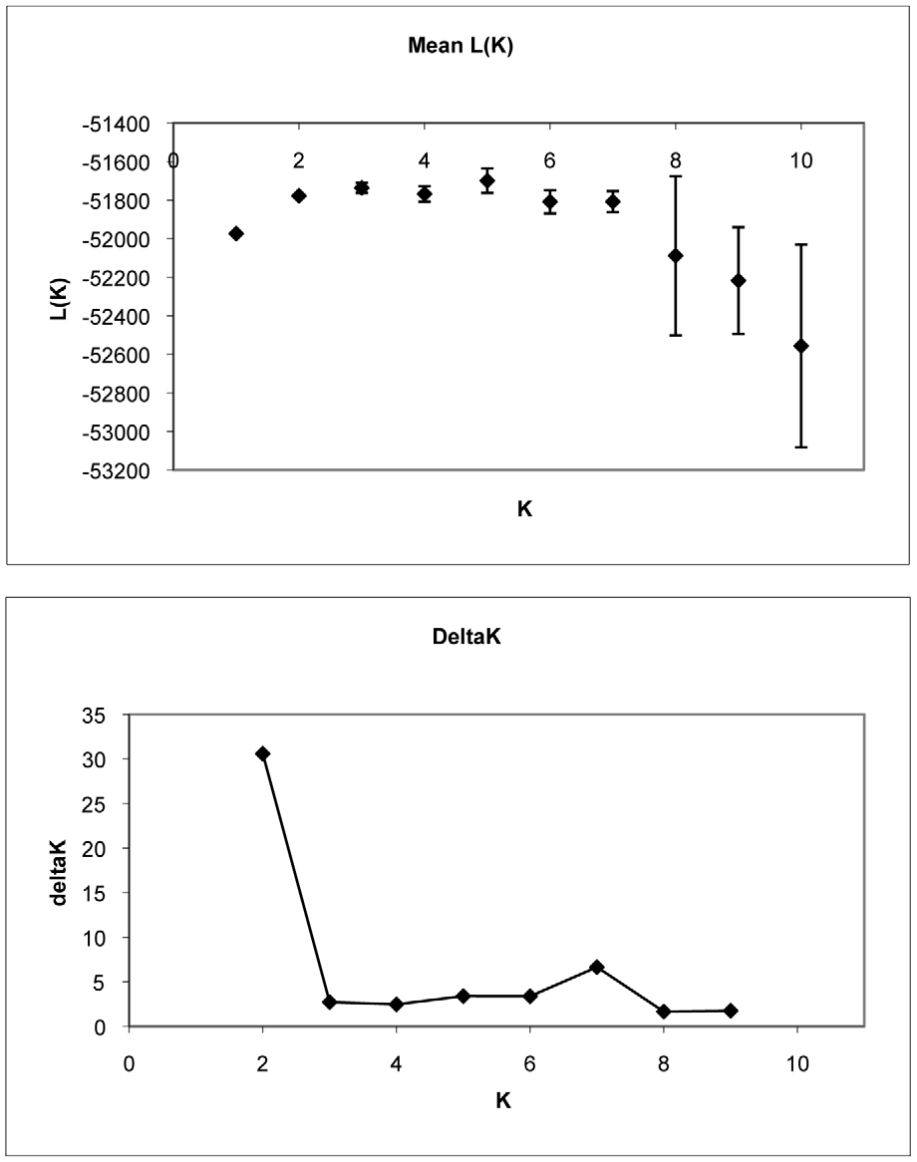
Likelihood values and ΔK calculations for STRUCTURE runs. Likelihood values obtained for 5 replicate runs of structure at K = 1 to K = 10. Mean and standard deviation across the five runs is provided. ΔK was calculated using the method outlined in [65].

**Figure 2:**
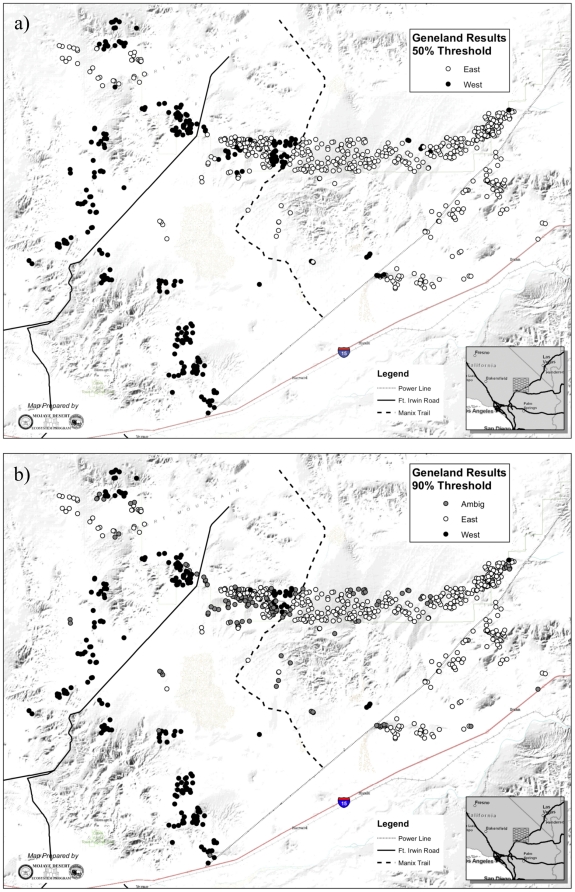
Results of GENELAND analysis, for a) 50% threshold and b) 90% threshold. Black circles indicate individuals assigned with ≥50% (or ≥90%) probability of membership to the ‘West’ subpopulation, and white circles indicate individuals assigned with ≥50% (or ≥90%) probability of membership to the ‘East’ subpopulation.

**Figure 3:**
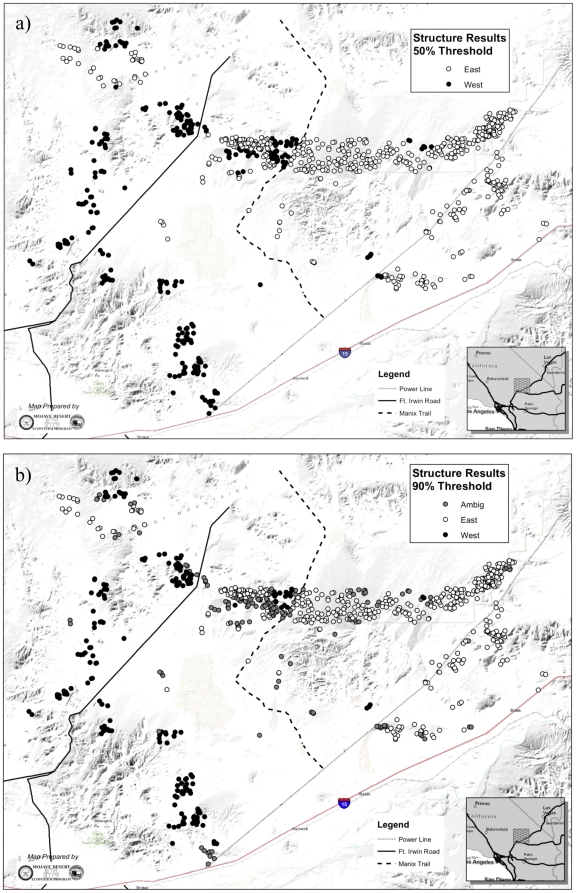
Results of STRUCTURE analysis, for a) 50% threshold and b) 90% threshold. Black circles indicate individuals assigned with ≥50% (or ≥90%) probability of membership to the ‘West’ subpopulation, and white circles indicate individuals assigned with ≥50% (or ≥90%) probability of membership to the ‘East’ subpopulation.

Clustering analysis revealed that desert tortoises were structured into two genetically distinct subpopulations. The most likely number of subpopulations in the dataset was two for both geneland and structure, although *K* = 1 was only slightly less likely using structure (Figures 1, 2, 3). This result was not unexpected, as structure does not always detect distinct subpopulations in cases where genetic differentiation between them is weak [31]. This limitation was somewhat alleviated by the high power afforded by our microsatellite marker set (unbiased probability of identity (P_ID_) = 1.9×10^−10^; [32]). There was a high level of agreement between the two clustering algorithms in terms of assignment of individual tortoises to the two subpopulations, despite the fact that the programs utilize different statistics to describe population membership (structure calculates *q*, the proportion of an individual's sampled genome that is characteristic of each subpopulation, and thus allows for individuals to be of mixed ancestry, whereas geneland calculates the probability that an individual belongs to each subpopulation; Figures 2 and 3).

The total population exhibited high levels of genetic variation. The number of alleles per locus ranged from 4 to 22, and of the 16 loci, 13 had at least 9 alleles and 9 had at least 18 alleles. The two subpopulations, denoted ‘East’ and ‘West’, exhibited weak but significant genetic differentiation (F_ST_ = 0.0046, p = 0.002), even when adjusted for the high heterozygosity of the microsatellites used (G′_ST_ = 0.00931). This low level of genetic differentiation between subpopulations indicates either a recent separation between these two subpopulations or a high level of ongoing gene flow between them. As expected for weakly differentiated subpopulations, standard genetic diversity measures for each subpopulation were similar (Table 1). Individuals that were not assigned to either subpopulation using the more stringent assignment threshold (90%) indicate that they are of mixed or ambiguous ancestry. These individuals were scattered throughout the landscape, rather than being concentrated along a zone of contact, indicating weak genetic differentiation across the landscape (Figures 2b and 3b).

**Table 1:** Genetic diversity estimates for inferred subpopulations and the total population.

Population	N	A	A_R_	H_e_	F_IS_
Total	859	14.4	13.5	0.738	0.043 (p<0.0001)
East	572	13.8	13.2	0.735	0.044 (p<0.0001)
West	287	13.6	13.5	0.738	0.041 (p<0.0001)

N, sample size; A, mean number of alleles per locus; A_R_, allelic richness; H_e_, expected heterozygosity; F_IS_, inbreeding coefficient. P values are given in parentheses.

Genetic distance between individuals (*a_r_*) was correlated with elevation (r = 0.008, p = 0.009), roads (Manix Trail r = 0.014, p = 0.00009; Fort Irwin Road r = 0.0237, p = 0.00001) and slope (r = 0.0394, p = 0.00001; Table 2). Given that elevation, roads and slope all were significantly related to genetic distance, we used partial Mantel tests to remove the influence of each variable while correlating the other with genetic distance. There was no significant relationship between genetic distance and elevation once the road- or slope-based correlation was removed. This suggests that elevation was a nuisance variable and not the main factor influencing gene flow. In contrast, the relationship between genetic distance and roads was always significant when slope was partialled out of the relationship. Likewise, the relationship between genetic distance and slope remained significant when the effect of roads was removed (Table 2). Mantel tests suggested no correlation between genetic distance and geographic distance (r = 0.004, p = 0.144), power lines (r = 0.007, p = 0.203), or soil type (r = 0.006, p = 0.013; Table 2).

**Table 2:** Mantel test results comparing matrices of genetic distance, topographic distance, elevation, slope, and position relative to roads and power lines among all pairs of individuals.

Correlation	Partialled out	Mantel *r*	*P*
*a_r_* 1 x Distance	—	0.0044	0.14400
*a_r_* x Elevation	—	0.0075	0.00898
*a_r_* x Manix Trail	—	0.0141	0.00009
*a_r_* x Fort Irwin Rd	—	0.0237	0.00001
*a_r_* x Power Lines	—	0.0070	0.20300
*a_r_* x Slope	—	0.0394	0.00001
*a_r_* x Soils	—	0.0056	0.01289
*a_r_* x Elevation	Manix Trail	0.0053	0.05150
*a_r_* x Elevation	Fort Irwin Rd	0.0003	0.46300
*a_r_* x Elevation	Slope	0.0050	0.05395
*a_r_* x Manix Trail	Elevation	0.0131	0.00030
*a_r_* x Manix Trail	Slope	0.0115	0.00102
*a_r_* x Fort Irwin Rd	Elevation	0.0225	0.00001
*a_r_* x Fort Irwin Rd	Slope	0.0203	0.00001
*a_r_* x Slope	Elevation	0.0390	0.00001
*a_r_* x Slope	Manix Trail	0.0386	0.00001
*a_r_* x Slope	Fort Irwin Rd	0.0375	0.00001

1 The measure of genetic distance used was Rousset's *a_r_* inter-individual distance (Rousset 2000).

The spatial genetic autocorrelation analysis revealed small, positive genetic correlation values for only the first distance class, and values not significantly different from zero at larger distance classes. We did not see a clear shift toward negative r-values at larger distance class, but instead observed a slight decreasing trend of r-values with increasing distance classes. Although it is possible that a large effective population size could be obscuring an isolation by distance pattern [33], our results suggest an overall lack of isolation by distance in this population.

## Discussion

Desert tortoises in our study area exhibit weak genetic structure at a local scale. We identified two genetically differentiated subpopulations within an apparently contiguous population. A small but significant level of genetic differentiation among the two subpopulations and similar levels of genetic diversity within subpopulations suggest that gene flow is restricted only slightly, or that the subpopulations were separated recently. Given the extremely long generation time for desert tortoises (20–25 years; [34]), ‘recent’ separation of subpopulations could have occurred dozens of years ago. Similarly, gene flow sufficient to maintain a low level of differentiation among subpopulations could be much less than one migrant per year or even one migrant every few decades in this species. This low level of population differentiation is consistent with other studies of desert tortoise in geographic areas of similar size [26].

Regardless of whether the separation between subpopulations is temporally or spatially slight, it is likely that some environmental feature (or suite of features) is restricting local movements of the desert tortoise. Barriers to gene flow that are relevant over ecological time can be difficult to detect because genetic divergence may be low [35], [36], [37]. In our study, thorough sampling (of both individuals and loci) at a local scale permitted detection of biologically relevant barriers to gene flow in spite of the fact that these barriers have resulted in only a very small departure from panmixia. These barriers warrant additional investigation to determine their continuing impact on desert tortoise movements. However, we urge caution in the interpretation of these results for conservation and management to avoid over-emphasizing the ecological significance of such small effects.

Our landscape genetic analysis identified both natural (slope) and anthropogenic (roads) landscape variables that have significantly influenced gene flow within this local population. The higher correlation of genetic distance with slope compared to roads indicates that gene flow is influenced predominantly by the slope of the landscape. Slope has been found as a barrier to gene flow in a limited number of other species, mostly pond-breeding amphibians [7], [38], [39], [40], [41], and indicates that gene flow is impeded by steep slopes. Accidental falls have been identified as a potentially important source of mortality [42] and could be serving as an effective barrier to gene flow over steep slopes. Alternatively, habitat preferences may influence desert tortoise movements and gene flow with respect to slope. In desert habitats, plant communities often differ drastically with slope due to variation in temperature, precipitation, evapotranspiration, and radiation at a micro scale. Desert tortoises are highly selective while foraging, selecting high quality plants and plant parts (those with a high Potassium Excretion Potential Index; [43]). These preferences may be even more pronounced in the western Mojave, where tortoises rely heavily on a peak of winter annual production for foraging.

Roads also influence desert tortoise movements. Desert tortoise pairs from the same side of a road exhibited significantly less genetic differentiation than tortoise pairs from opposite sides of a road. This pattern was found for both Manix Trail and Fort Irwin Road, despite differences in traffic volumes and construction materials. Both roads were constructed relatively recently (expanded to current size ∼1970 s), and it may seem that road construction is too recent to have impacted desert tortoise population structure. However, our results confirm simulation studies that suggest that new landscape features can have rapid effects on genetic structure that are detectable almost immediately [44]. The authors quantified the lag time to detect barriers in the landscape, and found a lag time of as little as one generation when using individual-based analyses, indicating that contemporary spatial genetic patterns can be caused by current landscapes [44]. This is particularly true for species such as desert tortoises, with high dispersal ability relative to the extent of the landscape and density of the population.

There are several possible ways in which roads could impact desert tortoises. First, roads may influence gene flow in desert tortoises directly via increased mortality during road crossings [45]. Second, roads may impact tortoises indirectly by providing increased human access to desert habitat, thereby magnifying anthropogenic impacts such as poaching and predation [46], [47], [48], [49]. Both of these mechanisms would serve to limit tortoise dispersal across roads, which would lead to an increase in genetic differentiation across the road as we observed. Both mechanisms also would be expected to yield a higher effect for Fort Irwin Road than for Manix Trail, a pattern consistent with our data. The third way in which roads could impact desert tortoises is by influencing burrowing behavior. Desert tortoises spend nearly 90% of their time in burrows, indicating that burrow placement is of particular importance in this species [50], [51]. Desert tortoises prefer steeply eroded banks of washes and areas with sufficient plant cover for burrow sites [52]. It is conceivable that roads provide habitat that is functionally similar to the washes and offer preferred burrow sites on either side of a road. Alternatively, plant productivity in the desert is often greater along roadsides (“edge-enhancement”; [53], [54], potentially offering preferred burrow sites [55]. The correlation between genetic distance and roads may therefore reflect an association with one or the other side of a road, and a propensity for tortoises to move along, rather than across, roads. Although these data may seem to be in direct contrast to the wealth of data documenting the negative impacts of roads on desert tortoises [29], [45], [56], other recent studies have also found desert tortoises in association with roads more often than expected [55], [57].

Our study indicates that both natural and anthropogenic features of the landscape influence patterns of genetic structure and gene flow in desert tortoises at a local scale. This is in contrast to patterns observed at a regional scale, where geographic distance plays a major role in shaping patterns of genetic structure [24], [25], [26], [27], [28]. In a recent landscape genetic study of desert tortoises, [28] identified elevation as a potential barrier to gene flow at a regional scale, and found no influence of slope (as part of a landscape resistance model). Although apparently in direct contrast to our results, it demonstrates that different landscape variables influence movements at different temporal and spatial scales [58], [59].

Although genetic structure in this population is currently affected by slope more than by roads, the extremely short time lag between the emergence of roads as a barrier and detection of a genetic effect indicates that roads may become increasingly important in shaping the evolutionary trajectory of desert tortoise populations. In addition, our results empirically support recent research suggesting that landscape effects on population structure may be limited in scale [16], but detectable almost immediately [44]. This is encouraging for those interested in characterizing the influences of recent landscape changes on fine-scale genetic structure and gene flow. The ability to detect barriers to gene flow almost immediately is also important for agencies and individuals working on endangered species management methods such as translocation, captive breeding and release, and reserve and corridor design. However, we urge caution in the interpretation of such data, as our results also demonstrate that barriers can be detected even when their effect on gene flow is relatively weak.

## Methods

### Ethics Statement

Permits to collect blood samples and transfer them to the laboratory included US Fish and Wildlife Service permit TE-102235-3 and California Scientific Collecting Permit SCP 801179-015.

### Sample Collection

Blood samples were collected from desert tortoises in the Southern Expansion Area (SEA) of the National Training Center in Fort Irwin, CA, and within the Southern Translocation Area (STA) to the south of Fort Irwin. The SEA is an area of approximately 9,700 ha that was systematically searched for desert tortoises during a clearance operation to completely remove desert tortoises in this area for subsequent translocation to the STA. All desert tortoises found (the entire population) were sampled. In the STA, samples were collected opportunistically. Desert tortoises in this region are part of the western Mojave Recovery Unit [60]. Geographic location, sex, elevation, soil type, and slope (based on a 30-meter digital elevation model around each point, with 0 being flat and 90 vertical) were collected for each sample. The location of samples relative to three putative barriers was also noted. Barriers included two roads that run roughly north-south through the SEA and STA, and are flat relative to the surrounding landscape (i.e., not sloped or elevated): Fort Irwin Road, a paved road with relatively high traffic volume and Manix Trail, an unpaved dirt road with low traffic volume. The third barrier was a large power line corridor running southwest-northeast through the STA.

### Laboratory Methods

DNA was extracted from blood samples using the BioSprint 96 robotic workstation (Qiagen) and the manufacturer's protocol. Desert tortoises were genotyped at 16 microsatellite loci developed for desert and gopher (*G. polyphemus*) tortoises [61], [62], [63]. All 16 loci were amplified in 4 multiplex reactions (Multiplex A: GOA 1, 11, 22, 23; Multiplex B: GOA 3, 4, 6, 14; Multiplex C: GOA2, 8, 12, 13; Multiplex D: Goag 4, Goag7, GP30, GP61). Each reaction contained ∼20 ng genomic DNA, 3–12 pmol primer, 2× PCR buffer, 2 mM each dNTP, and 1 Unit of Taq DNA polymerase. Thermocycler conditions were: 2-min initial denaturation step at 95°C; 30 cycles of 30 s at 95°C, 30 s at the annealing temperature (multiplex A = 59°C, B = 58°C, C = 56°C, D = 54°C), and 2 min at 72°C; followed by a 45 min soak at 60°C. PCR products were separated using an ABI 3130 automated DNA sequencer and the data were analyzed using genemapper version 3.7. Repeated genotyping for quality control was performed for 9% of the samples in the dataset (n = 1,291 genotypes); 7 repeated genotypes (0.5%) exhibited dropout of the larger allele and were reconciled in the final dataset. The final dataset consisted of 859 desert tortoises with a complete suite of data: multilocus microsatellite genotype, geographic location, sex, elevation, slope, and soil type.

### Data Analysis

To infer the number of subpopulations in the dataset and to assign individual samples to these subpopulations, we employed two Bayesian clustering techniques, geneland (version 0.3; [64]) and structure (version 2.1; [3], [65]). In geneland, we first varied the number of populations from 1 to 6, using a matrix of genotypes, spatial coordinates for each individual, and 950,000 stored MCMC iterations (10,000,000 iterations, thinning = 10, burnin 50,000). Allele frequencies were drawn from independent Dirichlet distributions [3], as this model has been shown to perform better than the alternative model (F-model) [5]. We allowed for correlated allele frequencies, allowed for null alleles (recommended whether or not the dataset contains null alleles; [66], and an uncertainty of 10 m on spatial coordinates. This model was run 5 times to assess precision. Alternate values for uncertainty (1 m, 100 m, 1000 m), and number of stored iterations (450,000 and 1,950,000) did not significantly alter the results. Not allowing for null alleles in the model slightly reduced consistency across runs in inferring K and in individual assignment, an effect described by [66]. Individuals were assigned to subpopulations based on their probability of population membership, using thresholds for assignment of 50% (to assign all individuals in a subpopulation) and 90% (allowing samples with a probability of membership <90% in all populations to remain unassigned).

In structure, we performed 5 runs at each value of the fixed parameter *K* (the number of subpopulations, from *K* = 1 to *K* = 6). Each run consisted of 500,000 replicates of the MCMC after a burn-in of 100,000 replicates. We used the admixture model and allowed allele frequencies to be correlated. All other parameters were set to default values [67]. This configuration is thought to provide the best resolution in the case of potentially subtle population structure [31], [65]. We observed the common phenomenon that once the true K is reached, likelihoods for larger *K*s plateau and the variance among runs increase [67]. Thus, we also used a Δ*K* measure that has been proposed to alleviate this problem and provide a more robust estimate of *K* [68]. To assign individuals to subpopulations, we performed a final run (1,000,000 burn-in and 1,000,000 replicates) at the inferred K. Values of *q*, the proportion of an individual's sampled genome characteristic of each subpopulation, were used to assign individuals to subpopulations at both a 50% and 90% threshold for assignment.

We assessed genetic diversity within each subpopulation (as determined by the 50% threshold geneland assignment) and the divergence among subpopulations with several standard measures; the number of alleles per locus (A), expected heterozygosity (H_e_), and genetic variation among individuals within subpopulations (F_IS_) for each locus and the average multilocus value for each population. To compute F_ST_, we used the method of [69] as implemented in spagedi (version 1.2; [70], but we used random permutations (n = 20,000) to assess significance rather than bootstrapping across loci. We also calculated a scaled measure of genetic differentiation, G′_ST_ [71] that accounts for the high level of heterozygosity inherent in microsatellite markers.

To assess the spatial pattern of genetic variation in desert tortoises, we performed a spatial autocorrelation analysis with genalex version 6.2 [72]. Analyses were based on matrices of pairwise inter-individual genetic distances and pairwise topographic distances. We used multilocus assessments to improve replication for a pair of individuals and provide a more precise analysis of genetic pattern [73], [74], [75], [76]. An important consideration for spatial autocorrelation analysis is the choice of distance classes because this choice can influence the outcome and interpretation [77], [78]. In our analyses, we adopted distance classes that allowed relatively even sample size per distance class. We also tested other distance class options and found they revealed similar patterns. For all analyses, the pattern had levelled out within 10 km, thus we present the results up to 10 km only. We tested for significant deviation of spatial autocorrelation patterns from the random distribution of genotypes by plotting the 95% confidence intervals for the null hypothesis (estimated by 1000 random permutations of individuals genotypes among geographic locations).

We used Mantel tests to determine correlational significance between matrices of inter-individual genetic distance (Rousset's *a_r_*; [79]) and several environmental factors: topographic distance, elevation, roads (including Manix Trail, which is unpaved and has low traffic volume, and Fort Irwin Road, which is paved and has high traffic volume), power lines, slope, and soil type. Pairwise distance matrices were calculated for topographic distance, elevation, and slope. For roads, power lines, and soil type, matrices were binary indicators of whether individuals were found on the same side (0) or opposite sides (1) of the putative barrier, or were sampled on the same (0) or different (1) soil types. Since only one of the two subpopulations (‘West’) contained tortoises on both sides of the power lines, we included only tortoises assigned to that subpopulation in comparisons involving the power lines. For all significant correlations, we used partial Mantel tests to determine whether correlations remained significant when the influence of other variables were removed. There has been debate regarding the use of partial Mantel tests, particularly with the use of randomizations to assess significance, which can be problematic when both variables are spatially non-random [80], [81], [82], [83]. However, this approach remains the most commonly used method for examining the association among distance matrices in population genetics (e.g., [15], [84]). In addition, recent work has shown that individual-based analyses such as Mantel tests are vastly more responsive than population-based approaches for detecting barriers to gene flow [44]. All Mantel and partial Mantel tests were performed using zt software [85]. Significance was assessed using 100,000 randomizations. We controlled for multiple tests using a false discovery rate procedure [86], [87], [88], a powerful alternative to the Bonferroni correction that seeks to minimize both type I and type II errors, with the allowed proportion of false positives set at 0.05.

## References

[1] Manel S, Schwartz MK, Luikart G, Taberlet P (2003). Landscape genetics: combining landscape ecology and population genetics.. Trends Ecol Evol.

[2] Storfer A, Murphy MA, Evans JS, Goldberg CS, Robinson S (2007). Putting the ‘landscape’ in landscape genetics.. Heredity.

[3] Pritchard JK, Stephens M, Donnelly P (2000). Inference of population structure using multilocus genotype data.. Genetics.

[4] Corander J, Walmann P, Sillanpaa MJ (2003). Bayesian analysis of genetic differentiation between populations.. Genetics.

[5] Guillot G, Estoup A, Mortier F, Cosson JF (2005). A spatial statistical model for landscape genetics.. Genetics.

[6] Chen C, Durand E, Forbes F, Francois O (2007). Bayesian clustering algorithms ascertaining spatial population structure: a new computer program and a comparison study.. Mol Ecol Notes.

[7] Funk WC, Blouin MS, Corn PS, Maxell BA, Pilliod DS (2005). Population structure of Columbia spotted frogs (*Rana luteiventris*) is strongly affected by the landscape.. Mol Ecol.

[8] Coulon A, Guillot G, Cosson JF, Angibault JMA, Aulagnier S (2006). Genetic structure is influenced by landscape features: empirical evidence from a roe deer population.. Mol Ecol.

[9] Latch EK, Scognamillo DG, Fike JA, Chamberlain MB, Rhodes OE (2008). Deciphering ecological barriers to North American river otter (*Lontra canadensis*) gene flow in the Louisiana landscape.. J Hered.

[10] Frantz AC, Pope LC, Etherington TR, Wilson GJ, Burke T (2010). Using isolation-by-distance-based approaches to assess the barrier effect of linear landscape elements on badger (*Meles meles*) dispersal.. Mol Ecol.

[11] Gerlach G, Musolf K (2000). Fragmentation of landscape as a cause for genetic subdivision in bank voles.. Conserv Biol.

[12] Riley SPD, Pollinger JP, Sauvajot RM, York EC, Bromley C (2006). A southern California freeway is a physical and social barrier to gene flow in carnivores.. Mol Ecol.

[13] Cushman SA, McKelvey KS, Hayden J, Schwartz MK (2006). Gene flow in complex landscapes: testing multiple hypotheses with causal modeling.. Am Nat.

[14] Wang IJ, Summers K (2010). Genetic structure is correlated with phenotypic divergence rather than geographic isolation in the highly polymorphic strawberry poison-dart frog.. Mol Ecol.

[15] Schwartz MK, Copeland JP, Anderson NJ, Squires JR, Inman RM (2009). Wolverine gene flow across a narrow climatic niche.. Ecology.

[16] Lee-Yaw JA, Davidson A, McRae BH, Green DM (2009). Do landscape processes predict phylogeographic patterns in the wood frog?. Mol Ecol.

[17] Wang IJ (2009). Fine-scale population structure in a desert amphibian: landscape genetics of the black toad (*Bufo exsul*).. Mol Ecol.

[18] Murphy RW, Berry KH, Edwards T, Leviton A, Lathrop A (2011). The dazed and confused identity of Agassiz's land tortoise, *Gopherus agassizii* (Testudines: Testudinidae) with the description of a new species and its consequences for conservation.. ZooKeys.

[19] U.S. Fish and Wildlife Service (USFWS) (1990). Endangered and threatened wildlife and plants; determination of threatened status for the Mojave population of the desert tortoise.. Fed Register.

[20] Lamb T, Avise JC, Gibbons JW (1989). Phylogeographic patterns in mitochondrial DNA of the desert tortoise (*Xerobates agassizii*), and evolutionary relationships among North American gopher tortoises.. Evolution.

[21] Germano DJ (1993). Shell morphology of North American tortoises.. Am Mid Nat.

[22] Lamb T, McLuckie AM, Van Devender TR (2002). Genetic differences among geographic races of the desert tortoise.. The Sonoran Desert Tortoise: Natural History, Biology and Conservation.

[23] Berry KH, Morafka DJ, Murphy RW (2002). Defining the desert tortoise(s): our first priority for a coherent conservation strategy.. Chelonian Conserv Biol.

[24] Murphy RW, Berry KH, Edwards T, McLuckie AM (2007). A genetic assessment of the recovery units for the Mojave population of the desert tortoise, *Gopherus agassizii*.. Chelonian Conserv Biol.

[25] Britten HB, Riddle BR, Brussard PF, Marlow R, Lee TE (1997). Genetic delineation of management units for the desert tortoise, *Gopherus agassizii*, in northeastern Mojave Desert.. Copeia.

[26] Edwards T, Goldberg CS, Kaplan ME, Schwalbe CR, Swann DE (2004). Implications of anthropogenic landscape change on inter-population movements of the desert tortoise (*Gopherus agassizii*).. Conserv Genet.

[27] Hagerty BE, Tracy CR (2010). Defining population structure for the Mojave desert tortoise.. Conserv Genet.

[28] Hagerty BE, Nussear KE, Esque TC, Tracy CR (2011). Making molehills out of mountains: landscape genetics of the Mojave desert tortoise.. Lands Ecol.

[29] Berry KH (1986). Desert tortoise (*Gopherus agassizii*) relocation: Implications of social behavior and movements.. Herpetologica.

[30] Walde AD, Walde AM, Delaney DK, Pater LL (2009). Burrows of desert tortoises (*Gopherus agassizii*) as thermal refugia for horned larks (*Eremophilia alpestris*) in the Mojave desert.. Southwestern Nat.

[31] Latch EK, Dharmarajan G, Glaubitz JC, Rhodes OE (2006). Relative performance of Bayesian clustering software for inferring population substructure and individual assignment at low levels of population differentiation.. Conserv Genet.

[32] Paetkau D, Waits LP, Clarkson PL, Craighead L, Vyse E (1998). Variation in genetic diversity across the range of North American brown bears.. Conserv Biol.

[33] Gauffre B, Estoup A, Bretangnolle V, Cosson JF (2008). Spatial genetic structure of a small rodent in a heterogeneous landscape.. Mol Ecol.

[34] U.S. Fish and Wildlife Service (USFWS) (1994). Desert Tortoise (Mojave Population) Recovery Plan.

[35] Waples RS (1998). Separating the wheat from the chaff: Patterns of genetic differentiation in high gene flow species.. J Hered.

[36] Hedrick PW (1999). Perspective: Highly variable loci and their interpretation in evolution and conservation.. Evolution.

[37] Waples RS, Gaggiotti O (2006). What is a population? An empirical evaluation of some genetic methods for identifying the number of gene pools and their degree of connectivity.. Mol Ecol.

[38] Spear SF, Peterson CR, Matocq MD, Storfer A (2005). Landscape genetics of the blotched tiger salamander (*Ambystoma tigrinum melanostictum*).. Mol Ecol.

[39] Lowe WH, Likens GE, McPeek MA, Buso DC (2006). Linking direct and indirect data on dispersal: isolation by slope in a headwater stream salamander.. Ecology.

[40] Giordano AR, Ridenhour BJ, Storfer A (2007). The influence of altitude and topography on genetic structure in the long-toed salamander (*Ambystoma macrodactylum*).. Mol Ecol.

[41] Richards-Zawacki C (2009). Effects of slope and riparian habitat connectivity on gene flow in an endangered Panamanian frog, *Atelopus varius*.. Divers Distrib.

[42] Riedle D, Averill-Murray RC, Grandmaison DD (2010). Seasonal variation in survivorship and mortality of desert tortoises in the Sonoran Desert, Arizona.. J Herpetology.

[43] Oftedal OT, Hillard S, Morafka DJ (2002). Selective spring foraging by juvenile desert tortoises (*Gopherus agassizii*) in the Mojave desert: Evidence of an adaptive nutritional strategy.. Chelonian Conserv Biol.

[44] Landguth EL, Cushman SA, Schwartz MK, McKelvey KS, Murphy M (2010). Quantifying the lag time to detect barriers in landscape genetics.. Mol Ecol.

[45] Boarman WI, Sazaki M, Evink G, Zeigler D, Garrett P, Berry J (1996). Highway mortality in desert tortoises and small vertebrates: success of barrier fences and culverts.. Trends in addressing transportation related wildlife mortality: Proceedings of the Transportation Related Wildlife Mortality Seminar.

[46] Stebbins RC (1974). Off-road vehicles and the fragile desert.. Amer Biol Teach.

[47] Bury RB, Luckenbach RA, Busack SD (1977). Effects of off-road vehicles on vertebrates in the California desert.. USFWS Wildl Res Report.

[48] Boarman WI (2002). Threats to desert tortoise populations: A critical review of the literature..

[49] Tracy CR, Averill-Murray R, Boarman W, Delehanty D, Heaton J (2004). Desert Tortoise Recovery Plan Assessment.. http://www.fws.gov/nevada/desert_tortoise/documents/dtrpac/dtrpac_report.pdf.

[50] Marlow RW (1979). Energy relations in the desert tortoise, *Gopherus agassizii*..

[51] Nagy KA, Medica PA (1986). Physiological ecology of desert tortoises.. Herpetologica.

[52] Luckenbach RA, Bury RB (1982). Ecology and management of the desert tortoise (*Gopherus agassizii*) in California.. North American Tortoises: Conservation and Ecology.

[53] Vasek FC, Johnson HB, Brum GD (1975). Effects of power transmission lines on vegetation of the Mojave Desert.. Madroño.

[54] Lightfoot DC, Whitford WG (1991). Productivity of creosotebush foliage and associated canopy arthropods along a desert roadside.. Am Mid Nat.

[55] Lovich JE, Daniels R (2000). Environmental characteristics of desert tortoise (*Gopherus agassizii*) burrow locations in an altered industrial landscape.. Chelonian Conserv Biol.

[56] Boarman WI, Sazaki M, Berry KH, Goodlett GO, Jennings WB (1993). Measuring the effectiveness of a tortoise-proof fence and culverts: status report from the first field season..

[57] Grandmaison DD, Ingraldi MF, Peck FR (2010). Desert tortoise microhabitat selection on the Florence Military Reservation, South-central Arizona.. J Herpet.

[58] Wiens J, Clobert J, Danchin E, Dhondt AA, Nichols JD (2001). The landscape concept of dispersal.. Dispersal.

[59] Holderegger R, Wagner HH (2008). Landscape genetics.. Bioscience.

[60] U.S. Fish and Wildlife Service (USFWS) (2008). Draft revised recovery plan for the Mojave population of the desert tortoise (*Gopherus agassizii*).

[61] Edwards T, Goldberg CS, Kaplan ME, Schwalbe CR, Swann DE (2003). PCR primers for microsatellite loci in the desert tortoise (*Gopherus agassizii*, Testudinidae).. Mol Ecol Notes.

[62] Schwartz TS, Osentoski M, Lamb T, Karl SA (2003). Microsatellite loci for the North American tortoises (genus *Gopherus*) and their applicability to other turtle species.. Mol Ecol Notes.

[63] Hagerty BE, Peacock MM, Kirchoff VS, Tracy CR (2008). Polymorphic microsatellite markers for the Mojave desert tortoise, *Gopherus agassizii*.. Mol Ecol Res.

[64] Guillot G, Mortier F, Estoup A (2005). geneland: a computer package for landscape genetics.. Mol Ecol Notes.

[65] Falush D, Stephens M, Pritchard JK (2003). Inference of population structure using multilocus genotype data: linked loci and correlated allele frequencies.. Genetics.

[66] Guillot G, Santos F, Estoup A (2008). Analysing georeferenced population genetics data with geneland: a new algorithm to deal with null alleles and a friendly graphical user interface.. Bioinformatics.

[67] Pritchard JK, Wen W (2003). Documentation for structure software: Version 2.. http://pritch.bsd.uchicago.edu.

[68] Evanno G, Regnaut S, Goudet J (2005). Detecting the number of clusters of individuals using the software structure: a simulation study.. Mol Ecol.

[69] Weir BS, Cockerham CC (1984). Estimating F-statistics for analysis of population structure.. Evolution.

[70] Hardy OJ, Vekemans X (2002). spagedi: a versatile computer program to analyze spatial genetic structure at the individual or population levels.. Mol Ecol Notes.

[71] Hedrick PW (2005). A standardized genetic differentiation measure.. Evolution.

[72] Peakall R, Smouse PE (2006). GenAlEx 6: genetic analysis in Excel. Population genetic software for teaching and research.. Mol Ecol Notes.

[73] Excoffier L, Smouse PE, Quattro JM (1992). Analysis of molecular variance inferred from metric distances among DNA haplotypes: application to human mitochondrial DNA restriction data.. Genetics.

[74] Peakall R, Smouse PE, Huff DR (1995). Evolutionary implications of allozyme and RAPD variation in diploid populations of dioecious buffalograss *Buchloe dactyloides*.. Mol Ecol.

[75] Smouse PE, Peakall R (1999). Spatial autocorrelation analysis of individual multiallele and multilocus genetic structure.. Heredity.

[76] Epperson BK (2004). Multilocus estimation of genetic structure within populations.. Theor Pop Biol.

[77] Peakall R, Ruibal M, Lindenmayer DB (2003). Spatial autocorrelation analysis offers new insights into gene flow in the Australian bush rat, *Rattus fuscipes*.. Evolution.

[78] Vekemans X, Hardy OJ (2004). New insights from fine-scale spatial genetic structure analyses in plant populations.. Mol Ecol.

[79] Rousset F (2000). Genetic differentiation between individuals.. J Evol Biol.

[80] Raufaste N, Rousset F (2001). Are partial Mantel tests adequate?. Evolution.

[81] Castellano S, Balletto E (2002). Is the partial Mantel test inadequate?. Evolution.

[82] Rousset F (2002). Partial Mantel tests: reply to Castellano and Balletto.. Evolution.

[83] Frantz AC, Do Linh San E, Pope LC, Burke T (2009). Using genetic methods to investigate dispersal in two badger (*Meles meles*) populations with different ecological characteristics.. Heredity.

[84] Walker FM, Taylor AC, Sunnucks P (2008). Female dispersal and male kinship-based association in southern hairy-nosed wombats (*Lasiorhinus latifrons*).. Mol Ecol.

[85] Bonnet E, Van de Peer Y (2002). zt: a software tool for simple and partial Mantel tests.. J Stat Software.

[86] Benjamini Y, Hochberg Y (1995). Controlling the false discovery rate: A practical and powerful approach to multiple testing.. J Royal Stat Soc B.

[87] Verhoeven KJF, Simonsen KL, McIntyre LM (2005). Implementing false discovery rate control: increasing your power.. Oikos.

[88] Narum SR (2006). Beyond Bonferroni: less conservative analyses for conservation genetics.. Conserv Genet.

